# Desmoid tumor of the anterolateral abdominal wall: A rare case report

**DOI:** 10.1016/j.amsu.2021.102804

**Published:** 2021-09-08

**Authors:** Mohamed Yassine Mabrouk, Laila Bouzayan, Samia Malki, Rachid Jabi, Amal Bennani, Mohammed Bouziane

**Affiliations:** a Department of General Surgery, Mohamed VI University Hospital, Oujda, Morocco; b Faculty of Medicine and Pharmacy, Laboratory of Anatomy, Microsurgery and Surgery Experimental and Medical Simulation (LAMCESM), Mohammed 1st University, Oujda, Morocco; c Department of Pathology, Mohammed VI University Hospital, Oujda, Morocco

**Keywords:** Desmoid tumor, Abdominal wall, Surgical excision, Parietal reconstruction, Case report

## Abstract

**Introduction and importance:**

Desmoid tumors of the abdominal wall are rare fibroblastic proliferative tissue, included in the group of soft tissue tumors, not metastasizing but locally aggressive as an infiltrating tumor and a source of recurrence.

**Case presentation:**

This case report describes a rare case of desmoid tumor of the anterolateral abdominal wall presented with a large mass of the left flank and iliac fossa. The diagnostic was suspected radiologically following an abdominopelvic computed tomography (CT) and magnetic resonance imaging (MRI). An exploratory laparotomy found a large mass infiltrating the left rectus abdominis muscle, a part of the flat left abdominal muscles, and the left iliac crest. The patient underwent a total resection of the mass involving the left rectus muscle with autoplasty by a pedicled flap of the left LATA fascia with the placement of a bifacial mesh.

Histologic analysis of the operatory specimen confirmed the diagnosis of a desmoid tumor of the abdominal wall.

The patient has been discharged from the hospital on the fifth day post-operatory with an uneventful recovery; she was in good health after a one-year follow-up.

**Clinical discussion:**

Desmoid tumors of the abdomen are very rare. Although this tumor is histologically benign, it has the potential of invading vital structures and has a high rate of local recurrence.

Histology staining confirms the diagnosis, surgery is the gold standard in the management of this pathology.

**Conclusion:**

We highlight the importance of radical surgical excision to avoid desmoid tumor complications and to minimize the recurrence risk.

## Highlights

• Desmoid tumor are rare benign tumor with potential of invading adjacent structures.
• The final diagnosis is made upon pathology study.
• Complete en-bloc resection is the gold standard treatement.
• The rate of recurrence is very high, and depends on the quality of the resection.

## 1 Introduction

Desmoid tumors, also known as aggressive fibromatoses, are part of the deep fibromatoses, which are themselves included in the group of soft tissue tumors, developed from connective tissue, fascia, or intramuscular walls [1].

Desmoid tumors are rare and represent less than 0.03% of all tumors and about 3.5% of fibrotic tumors [2], They are an infiltrant proliferation of the fibrotic tissue, which do not metastasize but they have a high local aggressive potential and a high tendency to recur [3] their diagnosis is often difficult, the clinical expression, sometimes late, is dominated by the appearance of a palpable tumor sometimes associated with pain, or by signs of compression. Certain radiological elements, such as computed tomography (CT), and magnetic resonance imaging (MRI), can provide competitive diagnostic data, but only histological examination provides a definitive diagnosis.

According to their location, desmoid tumors can be subdivided into an extra-abdominal tumor, intraabdominal tumor, and abdominal wall tumor [4]. Herein, we report a rare case of a desmoid tumor located in the abdominal wall in a female patient to which presents a particular interest for its diagnosis, treatment, and prognosis.

This work has been reported following SCARE 2020 guidelines [5].

## 2 Case presentation

A 41-year-old woman consulted to our for a painful mass in her left flank and left iliac fossa that has evolved for four months without fever, vomiting, nausea, or any symptom of gastrointestinal obstruction.

Her surgical past was remarkable by a Caesarean section one year ago.

She was a non-smoker with no drugs use, and had no history of allergies. The history of her family is unremarkable, as there is no similar case reported in the family or proven genetic abnormalities. The physical examination finds a soft and tender abdomen with the presence of a Pfannenstiel scar. Moreover, a mass occupying her left flank and left iliac fossa was palpated. The mass was non-compressible, freely mobile, and non-pulsatile.

**Fig. 1:**
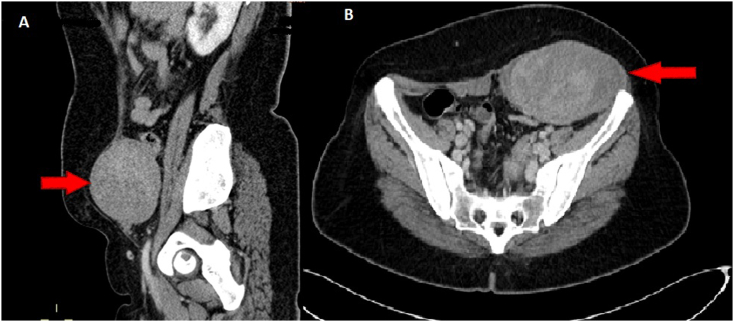
Abdominal CT scan showing a mass occupying the left anterolateral abdominal wall reaching the left iliac crest A: Coronal section B: Axial section.

**Fig. 2:**
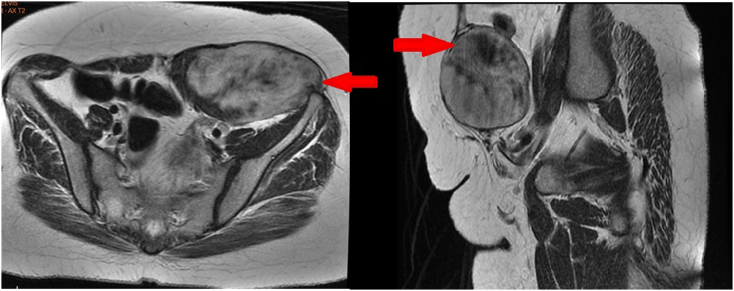
Abdominal MRI showing a mass located in the left abdominal wall reaching the left iliac crest measuring 11*6 cm with heterogeneous hyperintensity in T2-weighted.

To evaluate the abdominal mass detected by the physical examination. Abdominopelvic CT-scan was performed (Fig. 1) showing an ovoid mass suggestive of a desmoid tumor measuring 109*59 mm extended over 76 mm occupying the left anterolateral wall of the abdomen reaching the left iliac crest with intimate contact with the sigmoid loop, the abdominal MRI objectified a well-limited mass, with lobulated contours showing a T1 iso-intensity and a heterogeneous T2 hyperintensity, inflicting the rectus abdominis muscle and pushing outwards the left flat muscles of the abdomen as well as the iliacus muscle posteriorly and arriving at the contact of the left iliac crest below (see Fig. 2).

**Fig. 3:**
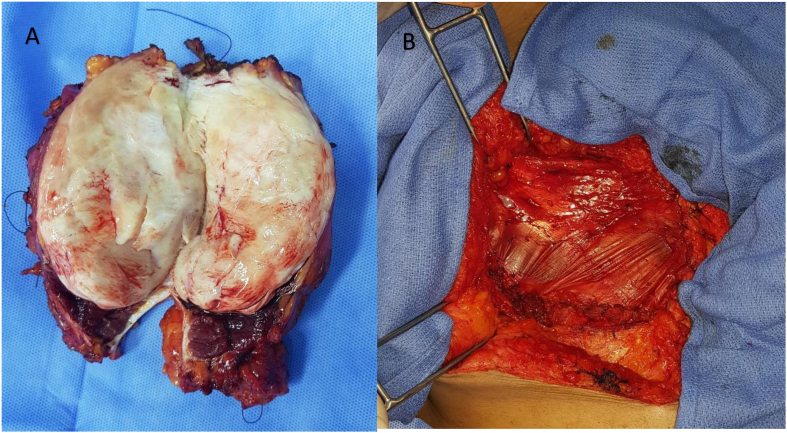
A: Image showing the resected mass. B: Interoperative image of reconstruction by a pediculed flap of the left fascia lata.

**Fig. 4:**
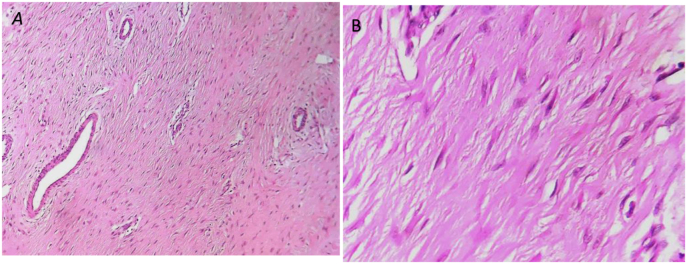
A: Microphotograph showing a homogeneous myofibroblastic proliferation made of long bundles, crossed by small vessels with arterialized walls (HE, x100). B: the tumor cells are not very atypical and are spaced from each other by a homogeneous collagenous frame (HE, x200). *HE:* hemotoxylin and eosin.

She underwent a midline laparotomy, intraoperative exploration revealed a parietal mass infiltrating the sub umbilical left rectus muscle, the left iliac crest, and the left anterosuperior iliac spine with adhesions to the greater omentum and the sigmoid colon. We decided to realize a complete excision of the mass for curative resection.

We proceeded to a total resection of the mass taking away the left rectus muscle and a part of the remaining muscle of the left abdomen wall with reconstruction by a pedicled flap of the left Fascia lata with the placement of a bifacial mesh with abdominal parietal drainage.(Figure 3).

The procedure was achieved by a chief of general surgery.

Postoperatively, analgesia, antibiotics, and prophylaxis for thromboembolism has been administered. The patient adhered to and tolerated the advice provided such as heavy lifting, and the use of abdominal support belt.

The histological examination found a fusocellular proliferation made of long and wide bundles that are inserted between the bundles of the striated muscle (in fingers of gloves). The tumor cells have elongated and sometimes undulating nuclei and eosinophilic cytoplasm with imprecise boundaries in favor of a desmoid tumor, the proliferation is based on an abundant keloid-like collagenous background which becomes focally lax.Figure 4

The patient's post-operative recovery was without incident, The drain was removed on the fourth postoperative day and she was discharged on the fifth postoperative day.

After a Follow up of one year after surgery, there was no evidence of recurrence.

## 3 Discussion

Desmoid tumors are rare mesenchymal tumors, accounting for approximately 3% of soft tissue sarcomas and 0.03% of all cancers [4]. The estimated incidence in the general population is 2–4 per million population per year [6].

They occur sporadically, as in the case of our patient, or in the context of Familial adenomatous polyposis (FAP), which is part of the Gardner syndrome [2]. The risk of developing a desmoid tumor in a patient with FAP is 852–1000 times greater than in the general population [7].

Desmoid tumors can occur between the ages of 5 and 80 years with a peak between 28 and 30 years with a median age of 28.5 years [8], These tumors are highly prevalent in pregnant patients [9].

Clinically, fibromatous tumors are usually painless, often revealed by the palpation of a mass of 3–10 cm, poorly limited, with little or no sensitivity, of slow and progressive growth without ever crossing the midline [3]. They pose problems during the closure of the wall in the case of a large tumor or during repeated operations for a recurrent tumor [3,10].

Desmoid tumors of the abdominal wall are particular in their location and in the circumstances in which they arise, they develop essentially in the rectus and oblique muscles. More rarely they may develop from the pelvic wall [11].

Large size free abdominal endometrioma can exibit the same clinical presentation with potential compression of the adjacent structure, it should be considered as a differential diagnosis of desmoid tumor [12].

Imaging relies mainly on CT and MRI which are useful for assessing the size and extension of desmoid tumors, studying surrounding structures, and evaluating tumor recurrence [13].

Ultrasound, the first-line examination, typically shows a hypoechoic mass, relatively well limited, smooth in its parietal location, and irregular and infiltrating in the other forms. The CT scan shows a more or less well-limited mass of tissue density, which is iso or discreetly hypodense comparing to the surrounding muscle, homogeneously enhancing, sometimes heterogeneous in large tumors with areas of necrosis, microhemorrhages, and metaplasia. MRI allows a better characterization of the tissue, a better study of the relationships with adjacent structures (nerves, vessels, deep organs), and the differentiation of postoperative changes or after medical treatment of a tumor recurrence [14]. It is often an ovoid or infiltrating mass, with generally lobulated or sometimes irregular boundaries, presenting a homogeneous iso or hypointensity in the T1-weighted sequence and a variable signal often in hyperintensity in the T2 sequence, the contrast gain is intense and heterogeneous after injection of gadolinium. The presence of bands in hypointensity, related to collagen bundles, on all sequences is very characteristic. A diameter greater than 10 cm, multiple mesenteric localization, invasion of the small intestine, or the superior mesenteric artery, and bilateral hydronephrosis are the factors of poor prognosis [14].

In our Case, CT and MRI scans were used to determine the exact location and relationship of the tumor which was the rectus abdominis and the flat muscles of the abdomen.

Curative surgery, when possible, should remain the treatment of choice despite the high risk of recurrence, the surgical excision must be large, passing through the healthy zone with a margin of a safety margin of 2–3 cm beyond the palpable tumor. However, complete resection cannot be judged macroscopically because the tumor is not encapsulated and the muscular extension is impractical [15].

The deliberately mutilating nature of the surgical resection and the parietal defects constitute a difficulty for the surgeon. Several procedures are used: directed cicatrization based on repetitive steps; fascial grafts known as auto genetic grafts, most often using the tensor fascia lata muscle; flaps using vascularized and pediculated structures.

This technique can use the dorsal major muscle, the fascia lata muscle, the rectus abdominis, the fascia lata, and the aponeurotic or epiploic flap.

The insertion of synthetic material must comply with the rules of rigorous asepsis, using resorbable or non-absorbable meshes whose insertion site may be subperitoneal, preperitoneal, or intraperitoneal [16].

In the case of non-resectability, medical treatment is based on hormonal therapy and non-steroidal anti-inflammatory drugs allowing stabilization or, more rarely, regression of the tumor size. Chemotherapy or external radiotherapy may be proposed in aggressive forms, resistant to medical treatment, or deemed untreatable as palliative or neo-adjuvant treatment [17].

In our case, the patient underwent a large excision with a parietal reconstruction using a fascia lata flap over a bifacial mesh.

The postoperative course was simple and no adjuvant treatment was used.

The histological study shows a monoclonal proliferation of spindle-shaped fibroblastic cells surrounded and separated from each other by collagen fibers arranged in bundles [18]. In our case, the diagnosis of a desmoid tumor was confirmed by a pathological study. Post-therapeutic monitoring is based on clinical examination and imaging, particularly MRI, which allows better visualization of local recurrences. These recurrences appear in the five years following surgery in 33–75% of cases and depend on the quality of excision, the growth phase at the time of surgical treatment, and the location and extension of the tumor [3].

Our patient underwent clinical and CT surveillance at 3 and 6 months with a 1-year follow-up and no locoregional recurrence was observed.

## 4 Conclusion

Desmoid tumors of the abdominal wall are rare, benign tumors with a high risk of local recurrence. The clinical manifestation of these tumors is characterized by masses that are often large. Complementary radiological examinations, in particular ultrasound, MRI and CT, allow the diagnosis to be suspected, but confirmation is only provided by anatomopathological examination, which reveals a proliferation of fibroblasts of benign appearance.

Treatment is ideally based on wide surgical which consists of carcinological removal of the tumor with sufficient safety margins followed by parietal reconstruction using prosthetic reinforcement if necessary. The combination of radiotherapy and hormonal therapy is a good alternative that provides objective answers in inoperable desmoid tumors and reduces the recurrence rate.

## Ethical approval

No ethical approval necessary.

## Sources of funding

The author(s) received no financial support for the research, authorship and/or publication of this article.

## Author statement

**Dr Mabrouk Mohamed Yassine:** Have written the article, have consulted the patient, prescribed all of the tests and prepared the patient for surgery and participated in the surgery.

**Dr Bouzayan Laila:** have helped writing the article, data collection.

**Dr Malki Samia:** Interpretation of histological data.

**Pr Benani Amal**(anatomopathology professor): confirm the histological diagnosis.

**Pr Jabi Rachid:** supervised the writing of manuscript.

**Pr Bouziane Mohammed** (oncology surgery professor): have supervised the writing of the paper, and has been the leader surgeon of the case.

## Trial registry number

Our paper is a case report; no registration was done for it.

## Guarantor

Mabrouk Mohamed Yassine.

## Consent

Written informed consent was obtained from the patient for publication of this case report and accompanying images. A copy of the written consent is available for review by the Editor-in-Chief of this journal on request.

## Declaration of competing interest

The authors declared no potential conflicts of interests with respect to research, authorship and/or publication of the article.
